# *catena*-Poly[tetra­sodium [[*cis*-dioxido-*trans*-bis­(sulfato-κ*O*)molybdate(VI)]-μ-sulfato-κ^2^ *O*:*O*′]]

**DOI:** 10.1107/S1600536808030328

**Published:** 2008-10-15

**Authors:** Susan J. Cline Schäffer, Rolf W. Berg

**Affiliations:** a Tårnby Gymnasium & HF, Tejn Allé 5, DK-2770 Kastrup, Denmark; b The Technical University of Denmark, The Department of Chemistry, Building 207, DK-2800 Lyngby, Denmark

## Abstract

Single crystals of the title compound, {Na_4_[Mo^VI^O_2_(SO_4_)_3_]}_*n*_, were grown from a melt of MoO_3_ and Na_2_SO_4_ in Na_2_S_2_O_7_. In contrast to the structure of the isoformular K compound, K_4_[Mo^VI^O_2_(SO_4_)_3_], with its monomeric anion, this sodium analogue contains a polymeric anion of the type {[Mo^VI^O_2_(SO_4_)_2_-μ-(SO_4_)]^4−^}_*n*_. The Mo^VI^ cations, surrounded by two tightly bonded O atoms and four O atoms of one bridging and two terminal sulfato ligands, form zigzag chains parallel to [100]. All four Na^+^ cations are situated between the anionic chains and have distorted octa­hedral coordination spheres.

## Related literature

The structure of the title isoformular potassium compound, K_4_[Mo^VI^O_2_(SO_4_)_3_], was determined by Schäffer & Berg (2008 ▶). For related Mo-containing compounds, see Salles *et al.* (1996 ▶) and Nørbygaard *et al.* (1998 ▶). Related compounds with Mo replaced by W were discussed by Schäffer & Berg (2005 ▶) and Berg *et al.* (2006 ▶). Other sulfato complexes coordinated to late transition metal centers were reported by Berg & Thorup (2005 ▶), Borup *et al.* (1990 ▶), Nielsen *et al.* (1993 ▶) and Rasmussen *et al.* (2003 ▶).
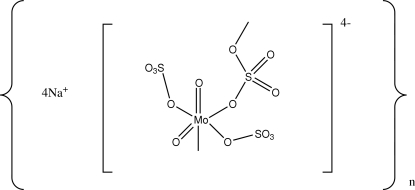


## Experimental

### 

#### Crystal data

Na_4_[MoO_2_(SO_4_)_3_]
*M* *_r_* = 508.08
Orthorhombic, 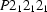

*a* = 8.4739 (6) Å
*b* = 9.2892 (7) Å
*c* = 15.1046 (11) Å
*V* = 1188.97 (15) Å^3^
*Z* = 4
Mo *K*α radiation
μ = 1.85 mm^−1^
*T* = 120 (2) K
0.24 × 0.18 × 0.02 mm

#### Data collection

Bruker SMART APEX CCD diffractometer
Absorption correction: gaussian (*SHELXTL*; Sheldrick, 2008 ▶) *T*_min_ = 0.665, *T*_max_ = 0.964
14125 measured reflections
2861 independent reflections
2817 reflections with *I* > 2σ(*I*)
*R*_int_ = 0.031

#### Refinement

*R*[*F* ^2^ > 2σ(*F* ^2^)] = 0.017
*wR*(*F* ^2^) = 0.042
*S* = 1.10
2861 reflections
199 parameters
Δρ_max_ = 0.51 e Å^−3^
Δρ_min_ = −0.26 e Å^−3^
Absolute structure: Flack (1983 ▶), 1205 Friedel pairs
Flack parameter: 0.01 (2)

### 

Data collection: *SMART* (Bruker, 2002 ▶); cell refinement: *SAINT* (Bruker, 2002 ▶); data reduction: *SAINT*; program(s) used to solve structure: *SHELXTL* (Sheldrick, 2008 ▶); program(s) used to refine structure: *SHELXTL*; molecular graphics: *SHELXTL*; software used to prepare material for publication: *SHELXTL*.

## Supplementary Material

Crystal structure: contains datablocks I, global. DOI: 10.1107/S1600536808030328/wm2186sup1.cif

Structure factors: contains datablocks I. DOI: 10.1107/S1600536808030328/wm2186Isup2.hkl

Additional supplementary materials:  crystallographic information; 3D view; checkCIF report

## Figures and Tables

**Table 1:** Selected bond lengths (Å)

Mo1—O2	1.6905 (16)
Mo1—O1	1.7108 (16)
Mo1—O5	1.9925 (16)
Mo1—O4	2.0102 (16)
Mo1—O6^i^	2.1661 (15)
Mo1—O3	2.1907 (15)

Symmetry code: (i) 
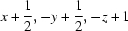
.

**Fig. 1.**
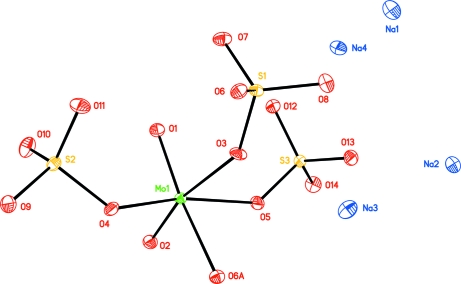
Plot of the asymmetric unit of Na4[MoVI(SO4)3O2], showing atoms as ellipsoids at the 50% probability level. [Symmetry code A) x+0.5, -y+0.5, -z+1.]

**Fig. 2.**
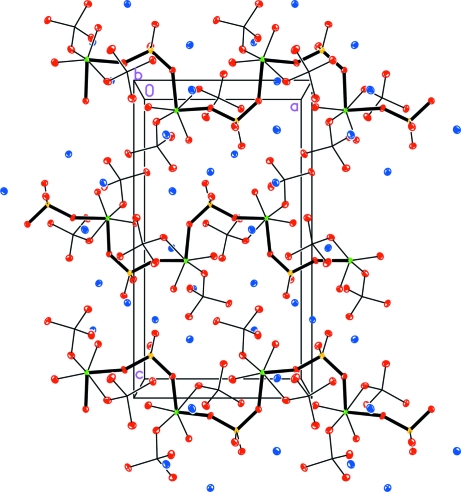
The crystal packing of Na4[MoVI(SO4)3O2], viewed along the b axis, showing the chains (thick black lines). Ellipsoids are displayed at the 50% probability level.

## supplementary crystallographic information

### Comment

Considerable amounts of molybdenum(VI) oxide, a solid well known for its insolubility in many acids, can be dissolved in sulfate melts at high temperatures, as was previously found for the chemically related tungsten(VI) oxide (Schäffer & Berg, 2005, Berg *et al.*, 2006). When varying molar amounts of MoO_3_, Na_2_SO_4_, and hygroscopic Na_2_S_2_O_7_ are placed in ampoules in a dry box, sealed, and heated to equilibration in a rocking furnace at 773 K for *ca.* 1 h, the resulting clear melts contain [MoO_2_]^2+^ moieties that are bonded to SO_4_^2-^ units. The compositions of the reaction products have been determined to be in the stoichiometric ratio 1:1:1, or MoO_3_ + *M*_2_SO_4_ + *M*_2_S_2_O_7_→*M*_4_[Mo(SO_4_)_3_O_2_]. In the case where *M* = K, a monomeric anion is formed (Schäffer & Berg, 2008), while for *M* = Na the anion is in a polymeric form.

The distorted octahedral coordination sphere of the Mo^VI^ cation contains two oxido ligands (*cis*), two terminally bound sulfato ligands (*trans*), and two O atoms of symmetry-related (*x* + 1/2, -*y* + 1/2, -*z* + 1) bridging sulfato ligands (*cis*), with O–Mo–O angles between any two *cis* oxygen atoms deviating as much as 15° from ideal values. The Mo–O bond distances to the tightly- bonded oxido ligands are similar (1.6905 (16) Å, 1.7108 (16) Å), which is expected as both bonds are *trans* to oxygen atoms in the bridging sulfato ligands. The Mo–O distances to the terminal sulfato ligands (Mo1–O4 and Mo1–O5) are slightly shorter than those to the brigding sulfato ligands, Mo1–03 and Mo1–O6A. The Mo–O distances compare well with previously reported values for related structures (Salles *et al.*, 1996; Nørbygaard *et al.*, 1998; Schäffer & Berg, 2008).

The coordination geometry of the sulfato ligands can be described as slightly distorted from tetrahedral, with angles ranging from 103.55 (9) to 113.77 (10)°. From the shortest to the longest, the S–O bond distances vary by type: S to terminal O atoms, 1.4516 (17)–1.4681 (17) Å; S to briding O atoms, 1.4941 (16) Å and 1.4953 (16) Å; S in the terminal sulfato ligands to the coordinating O atoms, 1.5589 (17) Å and 1.5346 (16) Å. This variation is typical for sulfato complexes of many different transition metal centers (Borup *et al.*, 1990; Nielsen *et al.*, 1993; Rasmussen *et al.*, 2003, and Berg & Thorup, 2005).

All four sodium cations are situated between the anionic chains and are six-coordinate with Na–O bond distances ranging from 2.2713 (18) to 2.7652 (18) Å.

### Experimental

Crystals were grown from a melt of equimolar amounts of MoO_3_, Na_2_SO_4_, and Na_2_S_2_O_7_, using a method described previously (Nørbygaard *et al.*, 1998).

### Refinement

On the basis of 1205 unmerged Friedel opposites, the fractional contribrution of the racemic twin was negligible (Flack, 1983). The two highest peaks in the final difference Fourier map were, respectively, 0.78 Å and 0.79 Å from Mo1, and the deepest hole was 1.31 Å from S2.

### Crystal data

**Table:**

Na_4_[Mo(SO_4_)_3_O_2_]	*F*(000) = 984
*M**_r_* = 508.08	*D*_x_ = 2.838 Mg m^−^^3^
Orthorhombic, *P*2_1_2_1_2_1_	Mo *K*α radiation, λ = 0.71073 Å
Hall symbol: P 2ac 2ab	Cell parameters from 5457 reflections
*a* = 8.4739 (6) Å	θ = 2.6–27.9°
*b* = 9.2892 (7) Å	µ = 1.86 mm^−^^1^
*c* = 15.1046 (11) Å	*T* = 120 K
*V* = 1188.97 (15) Å^3^	Tabular, colorless
*Z* = 4	0.24 × 0.18 × 0.02 mm

### Data collection

**Table:**

Bruker SMART APEX CCD diffractometer	2861 independent reflections
Radiation source: normal-focus sealed tube	2817 reflections with *I* > 2σ(*I*)
graphite	*R*_int_ = 0.031
ω scans	θ_max_ = 28.0°, θ_min_ = 2.6°
Absorption correction: gaussian (SHELXTL; Sheldrick, 2008)	*h* = −11→11
*T*_min_ = 0.665, *T*_max_ = 0.964	*k* = −12→12
14125 measured reflections	*l* = −19→19

### Refinement

**Table:**

Refinement on *F*^2^	Primary atom site location: structure-invariant direct methods
Least-squares matrix: full	Secondary atom site location: difference Fourier map
*R*[*F*^2^ > 2σ(*F*^2^)] = 0.017	*w* = 1/[σ^2^(*F*_o_^2^) + (0.0214*P*)^2^ + 0.3747*P*] where *P* = (*F*_o_^2^ + 2*F*_c_^2^)/3
*wR*(*F*^2^) = 0.042	(Δ/σ)_max_ = 0.001
*S* = 1.10	Δρ_max_ = 0.51 e Å^−^^3^
2861 reflections	Δρ_min_ = −0.26 e Å^−^^3^
199 parameters	Absolute structure: Flack (1983), 1205 Friedel pairs
0 restraints	Flack parameter: 0.01 (2)

### Special details

**Table:**

Geometry. All e.s.d.'s (except the e.s.d. in the dihedral angle between two l.s. planes) are estimated using the full covariance matrix. The cell e.s.d.'s are taken into account individually in the estimation of e.s.d.'s in distances, angles and torsion angles; correlations between e.s.d.'s in cell parameters are only used when they are defined by crystal symmetry. An approximate (isotropic) treatment of cell e.s.d.'s is used for estimating e.s.d.'s involving l.s. planes.
Refinement. Five frame series were filtered for statistical outliers then corrected for absorption by integration using *SHELXTL*/*XPREP* (Bruker, 2001) before using *SAINT*/*SADABS* (Bruker, 2002) to sort, merge, and scale the combined data. A series of identical frames was collected twice during the experiment to monitor decay. No decay correction was applied. The systematic conditions suggested the uambiguous space group. The structure was solved by direct methods (Sheldrick, 2001). The final space group choice was confirmed by successful convergence of the full-matrix least-squares refinement on *F*^2^. An extinction correction was not applied. The two highest peaks in the final difference Fourier map were, repectively, 0.78Å and 0.79Å from Mo1; the deepest hole was 1.31Å from S2. The final map had no other significant features. A final analysis of variance between observed and calculated structure factors showed no dependence on amplitude or resolution.

### Fractional atomic coordinates and isotropic or equivalent isotropic displacement parameters (Å^2^)

**Table:**

	*x*	*y*	*z*	*U*_iso_*/*U*_eq_	
Mo1	0.77389 (2)	0.108886 (19)	0.426923 (11)	0.00683 (5)	
S1	0.42167 (6)	0.29669 (6)	0.38157 (3)	0.00770 (11)	
S2	0.52730 (7)	−0.14389 (6)	0.48393 (3)	0.00870 (11)	
S3	0.87553 (6)	0.37753 (6)	0.29746 (3)	0.00819 (10)	
Na1	0.20072 (11)	0.58268 (10)	0.34042 (6)	0.01293 (19)	
Na2	0.80653 (11)	0.75841 (10)	0.31656 (6)	0.01239 (19)	
Na3	0.67303 (11)	0.51270 (10)	0.46834 (6)	0.0144 (2)	
Na4	0.57005 (11)	0.46216 (10)	0.21305 (6)	0.01274 (19)	
O1	0.70335 (19)	0.03236 (17)	0.33180 (10)	0.0107 (3)	
O2	0.94651 (19)	0.02131 (17)	0.44263 (11)	0.0119 (3)	
O3	0.57359 (18)	0.25797 (17)	0.42577 (11)	0.0107 (3)	
O4	0.6456 (2)	−0.02040 (17)	0.50576 (10)	0.0116 (3)	
O5	0.88450 (19)	0.28237 (18)	0.38036 (10)	0.0120 (3)	
O6	0.29354 (19)	0.27689 (17)	0.44854 (9)	0.0096 (3)	
O7	0.3902 (2)	0.20606 (18)	0.30489 (10)	0.0123 (3)	
O8	0.4313 (2)	0.44896 (18)	0.35609 (11)	0.0124 (3)	
O9	0.49519 (19)	−0.20813 (17)	0.56967 (11)	0.0130 (3)	
O10	0.60611 (19)	−0.24259 (18)	0.42327 (12)	0.0147 (3)	
O11	0.38958 (19)	−0.07781 (18)	0.44282 (11)	0.0139 (3)	
O12	0.7888 (2)	0.29908 (17)	0.22934 (10)	0.0124 (3)	
O13	0.7899 (2)	0.50790 (17)	0.32281 (10)	0.0126 (3)	
O14	1.03833 (19)	0.40786 (18)	0.27218 (10)	0.0133 (3)	

### Atomic displacement parameters (Å^2^)

**Table:**

	*U*^11^	*U*^22^	*U*^33^	*U*^12^	*U*^13^	*U*^23^
Mo1	0.00576 (8)	0.00737 (9)	0.00738 (8)	0.00034 (7)	−0.00028 (6)	−0.00021 (7)
S1	0.0056 (2)	0.0096 (3)	0.0078 (2)	0.0014 (2)	−0.00017 (19)	−0.0001 (2)
S2	0.0073 (2)	0.0093 (2)	0.0095 (2)	−0.0009 (2)	0.0006 (2)	−0.00087 (19)
S3	0.0073 (2)	0.0087 (2)	0.0086 (2)	−0.0001 (2)	0.00067 (18)	0.0004 (2)
Na1	0.0125 (5)	0.0130 (5)	0.0133 (4)	0.0022 (4)	0.0012 (3)	0.0019 (3)
Na2	0.0111 (5)	0.0126 (4)	0.0135 (4)	0.0012 (4)	0.0018 (4)	0.0017 (3)
Na3	0.0123 (4)	0.0166 (5)	0.0145 (5)	−0.0034 (4)	0.0023 (4)	−0.0024 (4)
Na4	0.0107 (4)	0.0156 (5)	0.0120 (4)	0.0010 (4)	−0.0008 (4)	0.0020 (4)
O1	0.0097 (8)	0.0119 (8)	0.0104 (7)	0.0002 (6)	−0.0002 (6)	−0.0008 (6)
O2	0.0113 (8)	0.0112 (8)	0.0130 (8)	0.0020 (6)	−0.0021 (6)	−0.0007 (6)
O3	0.0086 (7)	0.0128 (7)	0.0107 (7)	0.0044 (6)	−0.0029 (7)	−0.0008 (7)
O4	0.0138 (8)	0.0116 (8)	0.0095 (7)	−0.0053 (7)	−0.0018 (6)	0.0001 (6)
O5	0.0116 (8)	0.0121 (8)	0.0122 (8)	−0.0036 (6)	−0.0020 (6)	0.0042 (6)
O6	0.0080 (7)	0.0110 (7)	0.0098 (7)	−0.0010 (6)	0.0006 (6)	−0.0012 (6)
O7	0.0116 (8)	0.0162 (9)	0.0092 (7)	0.0016 (7)	−0.0016 (6)	−0.0028 (6)
O8	0.0121 (8)	0.0111 (8)	0.0140 (8)	0.0018 (7)	0.0023 (7)	0.0023 (6)
O9	0.0141 (8)	0.0131 (8)	0.0119 (7)	−0.0042 (6)	−0.0001 (7)	0.0028 (7)
O10	0.0151 (8)	0.0119 (8)	0.0171 (8)	−0.0010 (7)	0.0051 (7)	−0.0045 (7)
O11	0.0093 (8)	0.0190 (9)	0.0133 (8)	0.0021 (7)	−0.0016 (6)	0.0017 (6)
O12	0.0141 (8)	0.0120 (8)	0.0109 (7)	−0.0002 (7)	−0.0015 (6)	−0.0011 (6)
O13	0.0106 (8)	0.0108 (8)	0.0163 (8)	0.0025 (7)	−0.0008 (7)	−0.0015 (6)
O14	0.0095 (8)	0.0167 (9)	0.0136 (7)	−0.0012 (7)	0.0042 (6)	0.0008 (7)

### Geometric parameters (Å, °)

**Table:**

Mo1—O2	1.6905 (16)	Na1—O9^iv^	2.4972 (19)
Mo1—O1	1.7108 (16)	Na1—O1^ii^	2.7652 (18)
Mo1—O5	1.9925 (16)	Na2—O13	2.3333 (18)
Mo1—O4	2.0102 (16)	Na2—O14^v^	2.3350 (19)
Mo1—O6^i^	2.1661 (15)	Na2—O10^vi^	2.3415 (19)
Mo1—O3	2.1907 (15)	Na2—O9^i^	2.3932 (19)
S1—O7	1.4564 (16)	Na2—O7^ii^	2.5262 (18)
S1—O8	1.4681 (17)	Na2—O1^vi^	2.7006 (19)
S1—O3	1.4941 (16)	Na2—S3^v^	3.3837 (11)
S1—O6	1.4953 (16)	Na3—O11^i^	2.3524 (19)
S2—O9	1.4516 (17)	Na3—O2^iv^	2.3649 (19)
S2—O11	1.4575 (17)	Na3—O13	2.4115 (18)
S2—O10	1.4581 (17)	Na3—O10^vi^	2.4398 (19)
S2—O4	1.5589 (17)	Na3—O3	2.5928 (19)
S3—O14	1.4590 (17)	Na3—O8	2.724 (2)
S3—O12	1.4595 (16)	Na4—O7^ii^	2.3065 (19)
S3—O13	1.4627 (16)	Na4—O12	2.407 (2)
S3—O5	1.5346 (16)	Na4—O11^ii^	2.4078 (19)
Na1—O12^ii^	2.2713 (18)	Na4—O8	2.4629 (19)
Na1—O8	2.3273 (19)	Na4—O1^ii^	2.5003 (19)
Na1—O14^iii^	2.3649 (19)	Na4—O13	2.5297 (19)
Na1—O4^iv^	2.4394 (18)		
			
O2—Mo1—O1	102.72 (8)	O11^i^—Na3—O3	129.03 (7)
O2—Mo1—O5	91.81 (7)	O2^iv^—Na3—O3	75.84 (6)
O1—Mo1—O5	101.77 (7)	O13—Na3—O3	83.71 (6)
O2—Mo1—O4	95.58 (7)	O10^vi^—Na3—O3	134.87 (6)
O1—Mo1—O4	93.46 (7)	O11^i^—Na3—O8	175.92 (7)
O5—Mo1—O4	161.18 (7)	O2^iv^—Na3—O8	73.41 (6)
O2—Mo1—O6^i^	92.71 (7)	O13—Na3—O8	74.78 (6)
O1—Mo1—O6^i^	163.68 (7)	O10^vi^—Na3—O8	81.61 (6)
O5—Mo1—O6^i^	82.77 (6)	O3—Na3—O8	53.34 (5)
O4—Mo1—O6^i^	79.62 (6)	O7^ii^—Na4—O12	121.20 (7)
O2—Mo1—O3	167.36 (7)	O7^ii^—Na4—O11^ii^	90.91 (6)
O1—Mo1—O3	89.16 (7)	O12—Na4—O11^ii^	83.88 (6)
O5—Mo1—O3	81.39 (6)	O7^ii^—Na4—O8	102.80 (7)
O4—Mo1—O3	87.91 (6)	O12—Na4—O8	104.28 (6)
O6^i^—Mo1—O3	75.92 (6)	O11^ii^—Na4—O8	156.62 (7)
O7—S1—O8	111.01 (10)	O7^ii^—Na4—O1^ii^	81.21 (6)
O7—S1—O3	111.95 (9)	O12—Na4—O1^ii^	154.90 (7)
O8—S1—O3	107.53 (10)	O11^ii^—Na4—O1^ii^	84.66 (6)
O7—S1—O6	109.52 (9)	O8—Na4—O1^ii^	78.95 (6)
O8—S1—O6	109.65 (10)	O7^ii^—Na4—O13	78.73 (6)
O3—S1—O6	107.08 (9)	O12—Na4—O13	58.10 (6)
O9—S2—O11	113.77 (10)	O11^ii^—Na4—O13	124.20 (7)
O9—S2—O10	112.83 (10)	O8—Na4—O13	77.59 (6)
O11—S2—O10	111.33 (10)	O1^ii^—Na4—O13	144.70 (7)
O9—S2—O4	103.55 (9)	Mo1—O1—Na4^vii^	131.27 (9)
O11—S2—O4	107.18 (10)	Mo1—O1—Na2^viii^	110.49 (7)
O10—S2—O4	107.52 (10)	Na4^vii^—O1—Na2^viii^	91.78 (6)
O14—S3—O12	112.83 (10)	Mo1—O1—Na1^vii^	128.10 (8)
O14—S3—O13	112.19 (10)	Na4^vii^—O1—Na1^vii^	93.53 (5)
O12—S3—O13	110.37 (10)	Na2^viii^—O1—Na1^vii^	89.08 (5)
O14—S3—O5	106.14 (9)	Mo1—O2—Na3^i^	147.82 (9)
O12—S3—O5	108.22 (9)	S1—O3—Mo1	145.42 (10)
O13—S3—O5	106.73 (10)	S1—O3—Na3	99.86 (8)
O12^ii^—Na1—O8	119.12 (7)	Mo1—O3—Na3	108.85 (6)
O12^ii^—Na1—O14^iii^	115.36 (7)	S2—O4—Mo1	131.46 (9)
O8—Na1—O14^iii^	99.58 (7)	S2—O4—Na1^i^	98.63 (8)
O12^ii^—Na1—O4^iv^	131.24 (7)	Mo1—O4—Na1^i^	127.05 (8)
O8—Na1—O4^iv^	86.40 (6)	S3—O5—Mo1	136.93 (10)
O14^iii^—Na1—O4^iv^	98.10 (6)	S1—O6—Mo1^iv^	125.67 (9)
O12^ii^—Na1—O9^iv^	82.33 (6)	S1—O7—Na4^vii^	129.39 (10)
O8—Na1—O9^iv^	141.19 (7)	S1—O7—Na2^vii^	125.94 (10)
O14^iii^—Na1—O9^iv^	98.72 (7)	Na4^vii^—O7—Na2^vii^	101.54 (7)
O4^iv^—Na1—O9^iv^	57.26 (6)	S1—O8—Na1	119.62 (10)
O12^ii^—Na1—O1^ii^	72.64 (6)	S1—O8—Na4	107.73 (9)
O8—Na1—O1^ii^	76.04 (6)	Na1—O8—Na4	106.56 (7)
O14^iii^—Na1—O1^ii^	69.21 (5)	S1—O8—Na3	95.05 (8)
O4^iv^—Na1—O1^ii^	155.81 (6)	Na1—O8—Na3	125.35 (7)
O9^iv^—Na1—O1^ii^	142.71 (6)	Na4—O8—Na3	100.15 (7)
O13—Na2—O14^v^	130.56 (7)	S2—O9—Na2^iv^	147.79 (11)
O13—Na2—O10^vi^	85.66 (6)	S2—O9—Na1^i^	99.36 (8)
O14^v^—Na2—O10^vi^	143.70 (7)	Na2^iv^—O9—Na1^i^	99.58 (7)
O13—Na2—O9^i^	79.43 (6)	S2—O10—Na2^viii^	139.66 (10)
O14^v^—Na2—O9^i^	98.75 (7)	S2—O10—Na3^viii^	121.13 (10)
O10^vi^—Na2—O9^i^	89.40 (7)	Na2^viii^—O10—Na3^viii^	91.56 (6)
O13—Na2—O7^ii^	78.32 (6)	S2—O11—Na3^iv^	119.33 (9)
O14^v^—Na2—O7^ii^	93.97 (6)	S2—O11—Na4^vii^	111.58 (9)
O10^vi^—Na2—O7^ii^	91.17 (7)	Na3^iv^—O11—Na4^vii^	128.97 (8)
O9^i^—Na2—O7^ii^	157.64 (7)	S3—O12—Na1^vii^	138.53 (11)
O13—Na2—O1^vi^	156.46 (7)	S3—O12—Na4	98.37 (8)
O14^v^—Na2—O1^vi^	70.79 (6)	Na1^vii^—O12—Na4	122.67 (8)
O10^vi^—Na2—O1^vi^	73.15 (6)	S3—O13—Na2	141.74 (10)
O9^i^—Na2—O1^vi^	109.81 (6)	S3—O13—Na3	117.23 (9)
O7^ii^—Na2—O1^vi^	91.70 (6)	Na2—O13—Na3	92.48 (6)
O11^i^—Na3—O2^iv^	110.07 (7)	S3—O13—Na4	93.14 (8)
O11^i^—Na3—O13	101.80 (6)	Na2—O13—Na4	100.69 (7)
O2^iv^—Na3—O13	148.13 (7)	Na3—O13—Na4	107.34 (7)
O11^i^—Na3—O10^vi^	95.79 (7)	S3—O14—Na2^ix^	124.60 (10)
O2^iv^—Na3—O10^vi^	95.42 (7)	S3—O14—Na1^x^	124.66 (10)
O13—Na3—O10^vi^	81.85 (6)	Na2^ix^—O14—Na1^x^	109.32 (7)

Symmetry codes: (i) *x*+1/2, −*y*+1/2, −*z*+1; (ii) −*x*+1, *y*+1/2, −*z*+1/2; (iii) *x*−1, *y*, *z*; (iv) *x*−1/2, −*y*+1/2, −*z*+1; (v) −*x*+2, *y*+1/2, −*z*+1/2; (vi) *x*, *y*+1, *z*; (vii) −*x*+1, *y*−1/2, −*z*+1/2; (viii) *x*, *y*−1, *z*; (ix) −*x*+2, *y*−1/2, −*z*+1/2; (x) *x*+1, *y*, *z*.
